# An integrated hierarchical Bayesian approach to normalizing left-censored microRNA microarray data

**DOI:** 10.1186/1471-2164-14-507

**Published:** 2013-07-26

**Authors:** Jia Kang, Ethan Yixun Xu

**Affiliations:** 1 Department of Biometrics Research, Merck Research Laboratories, Rahway, NJ 07065, USA; 2 Department of Safety Assessment, Merck Research Laboratories, West Point, PA 19486, USA; 3 Present Address: Discovery Informatics, Infinity Pharmaceuticals, 780 Memorial Drive, Cambridge, MA 02139, USA

**Keywords:** miRNA, Normalization, Hierarchical Bayesian modeling, Detection limit, Variable selection

## Abstract

**Background:**

MicroRNAs (miRNAs) are small endogenous ssRNAs that regulate target gene expression post-transcriptionally through the RNAi pathway. A critical pre-processing procedure for detecting differentially expressed miRNAs is normalization, aiming at removing the between-array systematic bias. Most normalization methods adopted for miRNA data are the same methods used to normalize mRNA data; but miRNA data are very different from mRNA data mainly because of possibly larger proportion of differentially expressed miRNA probes, and much larger percentage of left-censored miRNA probes below detection limit (DL). Taking the unique characteristics of miRNA data into account, we present a hierarchical Bayesian approach that integrates normalization, missing data imputation, and feature selection in the same model.

**Results:**

Results from both simulation and real data seem to suggest the superiority of performance of Bayesian method over other widely used normalization methods in detecting truly differentially expressed miRNAs. In addition, our findings clearly demonstrate the necessity of miRNA data normalization, and the robustness of our Bayesian approach against the violation of standard assumptions adopted in mRNA normalization methods.

**Conclusion:**

Our study indicates that normalization procedures can have a profound impact on the detection of truly differentially expressed miRNAs. Although the proposed Bayesian method was formulated to handle normalization issues in miRNA data, we expect that biomarker discovery with other high-dimensional profiling techniques where there are a significant proportion of left-censored data points (e.g., proteomics) might also benefit from this approach.

## Background

MicroRNAs (miRNAs) are small non-coding RNAs that are critical players in mediating post-transcriptional genes regulations. They predominantly suppress gene expression by binding to the their target mRNAs to form RNA-induced silencing complex (RISC) [1] and are responsible for regulating >60% of the human coding genome [2-4].

As miRNAs are increasingly implicated in a number of important physiological and pathological processes (e.g., developmental timing [5], stem cell differentiation [6], cancer initiation and progression [7],), there has been an immensely growing interest within the scientific community to study the functional roles of miRNAs. To date, more than 18,226 hairpin precursor miRNAs from 168 species have been registered in the miRBase (http://www.mirbase.org/), and this number is expected to grow much further.

Microarray is a widely adopted technology to simultaneously quantify the expression of hundreds of miRNAs in one single experiment. Although this technology has shown enormous scientific potential in the comprehension of genomic regulation processes, many sources of systematic variation may influence its measured probe expression levels. The purpose of microarray data normalization is to minimize the array-level systematic bias, such that meaningful biological comparisons can be made and true biological changes can be found within one and multiple experiments.

A number of normalization methods have been developed for mRNA arrays in the past. Normalization methods for mRNAs often rely on one or more of the following assumptions: only a small fraction of features are differentially expressed and the proportions of up- and down-regulated expressions are approximately equal. In addition, for normalization methods based on housekeeping genes, the measurements for a set of housekeeping genes, which are expected to be expressed at relatively invariant levels across different tissues or treatment conditions, need to be well defined.

However, in contrast to mRNA arrays, which have an exceedingly high density of probes that are *in situ* synthesized on the array; miRNA microarrays are often lower density spotted arrays [8]. In addition, it was found in previous studies that (1) differentially expressed miRNAs are not always symmetric in the directions of regulation [9] (2) the fraction of differentially expressed miRNAs among treatment conditions often exceed 15%, and can be as high as 38% [10-12]. (3) Most commercially available miRNA microarrays do not have controls for endogenous RNAs or miRNAs that have been proven to be robustly invariant among different tissues or treatment conditions. Furthermore, different from mRNA arrays, where the abundance level for most of the probes is above the detection limit (DL) of the instrument; in miRNA arrays, we often have a large portion of miRNAs that are expressed below DL [13], resulting in the type of missing data called “non-ignorable missing”, and the simple exclusion of such “interval-censored” data can significantly bias results [14].

Given the differences between miRNA and mRNA data, it is questionable whether the normalization methods for mRNA arrays are adequate for miRNA arrays. In light of developing a more robust normalization method for miRNA arrays, Suo et al. [15] recently proposed a least-variant set (LVS) approach based on a set of data-driven invariant miRNAs, and demonstrated its superiority to other normalization methods including quantile normalization using a set of human tissue miRNA profiling data. However, LVS implicitly assumes the existence of a set of invariant miRNAs, which in reality may not hold. In addition, LVS is not designed to appropriately handle the missing data problem found in miRNA datasets as described previously.

The framework of Bayesian Hierarchical Modeling (BHM) has proved to be successful for analyzing many types of complex datasets in genetics. Using a BHM strategy has the following key benefits: (1) BHM allows the modeling of noise additively, multiplicatively, or in a nonlinear fashion; (2) Hierarchical modeling enables information borrowing from the comparable units to strengthen statistical inference when the dataset is sparse due to the usual small sample size of biological studies, such that genes exhibiting unusual small variability by chance will not lead to artificially inflated values of test statistics. (3) Bayesian framework offers a natural platform for handling data below detection limit. These benefits motivated us to develop a fully Bayesian approach to miRNA data normalization so that our new algorithm could perform missing data imputation, array normalization, and signal extraction under one unified framework.

Recently, several studies compared the performance of mRNA normalization methods on miRNA microarray data [8,15-17]. Rao et al. [8] and Zhao et al. [16] demonstrated that quantile normalization [18] (http://oz.berkeley.edu/~bolstad/stuff/qnorm.pdf) outperformed the other normalization methods they evaluated. In their studies, the primary objective was to compare the effect of normalization methods in reducing the variation among technical replicates. However, it is natural to expect that more aggressive normalization methods such as quantile normalization (e.g. forcing each array to have the exact same empirical distribution of intensities) will reduce variations among samples not only within the treatment group but also between treatment groups, which could make the discovery of truly differentially expressed features more difficult. Therefore, reduction of variation among technical replicates does not necessarily lead to an improved performance of feature selection, which is one of the most important goals of miRNA profiling experiments. To date, we are not aware of any literature that systematically compares the impact of different normalization methods in feature selection for the miRNA data. In this paper, we will compare the performance of Bayesian Normalization versus a few other widely adopted normalization methods in selecting differentially expressed (DE) miRNAs, through both a carefully designed simulation study and the analysis of a real dataset.

## Methods

We start with an ANOVA model for the log transformed miRNA expression *y*_*gcr*_ for miRNA *g*, experimental condition *c* (c = 1, 2), and condition-specific replicate *c*_*r*_, as

(1) $$\begin{array}{l} y_{g1r}∼N\left(a_{g}-\frac{1}{2}\delta_{g}+\beta_{g1r},\sigma^{2}g\right)\\ y_{g2r}∼N\left(a_{g}+\frac{1}{2}\delta_{g}+\beta_{g2r},\sigma^{2}g\right) \end{array}$$

suggested by Kerr et al [19]. We parameterize the mean of *y*_*gcr*_ to include additive effects at both miRNA and array levels, such that where *α*_*g*_ is the miRNA effect or overall expression level of miRNA *g*, *β*_*gcr*_ is the array effect that depends on *g* through *α*_*g*_, and *σ*^*2*^_*g*_ is the variance for miRNA *g*. We assume that the variance of each miRNA does not depend on the treatment conditions. The differential effect for each miRNA *g* between the two treatment conditions is denoted as *δ*_*g*._

To facilitate information borrowing among miRNAs with a Bayesian approach, we assume that *σ*^*2*^_*g*_ 's are exchangeable among miRNAs, and they come from a common distribution, which is chosen to be lognormal, following the notation of Hein et al [20].

(2) $$\sigma^{2}{}_{g}∼\mathrm{logNorm}\left(\mu ,\eta^{2}\right)$$

To capture the possibility that the array-level effect may impact different miRNAs of the same array differently, we model *β*_*gcr*_ as a linear function of *α*_*g*_, where $a^{0}{}_{\mathrm{cr}}$ and $a^{1}{}_{\mathrm{cr}}$ are regression coefficients.

(3) $$\beta_{\mathrm{gcr}}=a^{0}{}_{\mathrm{cr}}*a_{g}+a^{1}{}_{\mathrm{cr}}$$

the model is made identifiable by normalizing within each condition by setting ${\bar{\beta }}_{\mathrm{gc}.}=0$∀g,c, where the dot indicates that we are taking an average over the index *r*.

### Handling measurements below level of detection

One unique feature of miRNA data is that the expression level of miRNAs in different tissues or treatment conditions are often so low that they are below instruments' DL, resulting in a large portion of left-censored measurements. To reflect the left-censored nature of the observed data, we modify our likelihood specification for *y*_*gcr*_ such that

(4) $$y_{\mathrm{gcr}}∼N\left(a_{g}\pm \frac{1}{2}\delta_{g}+\beta_{\mathrm{gcr}},\sigma^{2}{}_{g}\right)I\left(L_{\mathrm{cr}},\right)$$

where *L*_cr_ is the level of detection for array *c*_*r*_ under condition *c*, which are readily available from the array manufacturers, and *I*(,) represents interval censoring, following the notation in JAGS^27^ software language.

### Winner’s curse correction

It is tempting to rank the features directly using the unadjusted posterior estimate of_*,*_ the absolute effect size for each miRNA (*δ*_*g*_ ), which is what Hein et al. adopted as their feature ranking criterion for mRNA microarray data [20]. However, in high dimensional genetic studies, estimates of effect sizes reported from the same discovery samples that were initially used to declare statistical significance are often grossly inflated and can not be replicated in an independent study. This type of bias is widely known as the Beavis effect [21] or the winner’s curse [22]. Prioritizing features with solely uncorrected *δ*_*g*_ is likely to generate many false findings.

The Bayesian paradigm allows us to conveniently correct for winner's curse by incorporating into our model the prior belief that the significance of the effect observed for each feature may be due to random chance. More mathematically, the effect size *δ*_*g*_ can be modeled with a mixture prior among a discrete probability with mass at zero, a continuous density f^+^ with support on the positive line, and a continuous density f^-^ with support on the negative line. This three-component mixture prior describes the three possible categories that a miRNA could be classified under: non-differentially expressed, up-regulated, or down-regulated, respectively.

More formally, we can model

(5) $$p\left(\delta_{g}\left|\xi_{1},\xi_{2}\right)\right)=\xi_{1}\delta_{\left\{0\right\}}\left(\delta_{g}\right)+\xi_{2}f^{+}\left(\delta_{g}\right)+\left(1-\xi_{1}-\xi_{2}\right)f^{-}\left(\delta_{g}\right)$$

We treat $\vec{\xi }$ as a hyperparameter with a Dirichelet distribution, i.e. $\vec{\xi }$ ~Dirichlet(α_1,_α_2,_α_3_). The parameters α_1,_α_2,_α_3_ reflect our degree of prior belief in *δ*_g_ = 0 (false positive) versus *δ*_g_ ≠ 0 (true positive).

As discussed previously, unlike mRNA datasets, where it has been well established that only a small fraction of features can be differentially expressed; for miRNA datasets, we could not favor, a priori, any region of (0,1) for $\vec{\xi }$, because the proportions of differentially expressed features among different miRNA datasets could vary dramatically. Therefore, we set α_1_=α_2_=α_3_=1, which essentially makes p($\vec{\xi }$ |α_1_=1_,_α_2_=1_,_α_3_=1) a uniform distribution over the simplex of possible values of $\vec{\xi }$.

We assume the non-null components (e.g. f^+^ and f^-^) of the mixture prior for *δ*_*g*_ to follow truncated normal distributions.

(6) $$\begin{array}{l} f^{+}\left(\delta_{g}\right)∼N\left(\mu^{+},\sigma^{{+}^{2}}\right)\left[\mathrm{truncated}\left(0,\infty \right)\right]\\ f^{-}\left(\delta_{g}\right)∼N\left(\mu^{-},\sigma^{{-}^{2}}\right)\left[\mathrm{truncated}\left(-\infty ,0\right)\right] \end{array}$$

### Additional prior specification

To complete the model, we assigned the following prior distributions for the remaining parameters. The gene effect *α*_*g*_*~N(G,S*^*2*^*)*, where *G~N(0,1000), S*^*-2*^*~Gamma(0.001,0.001)*.

The hyperparameters for miRNA level variance that appear in equation (2) are assumed to follow *μ~Ν(0,100),η*^*−2*^*~ Gamma(0.001,0.001).* The slope and intercept parameters that appear in equation (3) are assumed to follow: $a^{0}{}_{\mathrm{cr}}$*~N(0,100),*$a^{1}{}_{\mathrm{cr}}$*~N(0,100)*.

And finally, the hyperparameter for the non-null components of the effects size that appear in equation (6) are assume to follow: $\mu^{+}$ ~ N(0,100) [*truncated*(0, *∞*)], $\mu^{-}$ ~ N(0,100) [*truncated*(−*∞*, 0)], $\sigma^{+}{}^{-2}$*~ Gamma(0.001,0.001),*$\sigma^{{-}^{-2}}$*~ Gamma(0.001,0.001)*. Essentially, all of the priors and hyper-priors in our model were specified in a non-informative fashion.

### MCMC implementation

Each parameter was sampled from their posterior distributions by an MCMC algorithm using the JAGS software [23]. Specific parameter values and algorithm details not provided in this manuscript may be found in the R package BMIRN (Additional file 1). The instruction manual on how to use BMIRN can be found in Additional file 2.

On our Linux cluster (16 GB memory, and a dual-dual core 3.0 GHz AMD processor), where independent MCMC chains can be parallelized, it takes about 302 seconds on average to run 20,000 iterations for the size of data described in our simulation study (we find 20,000 iterations typically more than enough steps to achieve convergence in both simulation and the real data analysis in our study).

### Simulation studies

#### Study objective

The main objective of this simulation analysis is to explore how the unique features of miRNA datasets may impact the performance of the proposed Bayesian normalization method and the existing normalization methods.

Our investigation focuses on the following two aspects of the miRNA data that are different from the traditional mRNA data:

1. The proportion of differentially expressed features in miRNA data may be large.

2. The proportion of missing values caused by measurements below instrument's detection limit in miRNA data may be large.

#### Simulation setup

Following the general simulation setup of Broët et al. [24], we generate a microarray dataset containing 300 miRNAs and five repeat arrays under two conditions. The gene effects *α*_*g*_*'*s range uniformly between 0 and 10, and the array effects are linear functions of the gene effects. Following the notations described in equation (3), we generate $a^{0}{}_{\mathrm{cr}}$ from *N(0,0.5),*and $a^{1}{}_{\mathrm{cr}}$ from *N(0,0.05)* in our analysis. The gene variances are simulated based on equation (2), with *μ*=−1.8 and *η* =1, giving a similar range of variances to those we have observed in real data.

A sparsity parameter *sp* is created corresponding to the percentage of miRNAs in the simulated dataset that are truly differentially expressed. The differential effect $\delta_{g}$ for miRNA *g* is then set to be zero for the (1-*sp*) non-DE miRNAs, N(log(3),0.1^2^) for the $\frac{\mathrm{sp}}{2}$ up-regulated miRNAs, and N(−log(3),0.1^2^) for the remaining down-regulated $\frac{\mathrm{sp}}{2}$ miRNAs. In our simulation, *sp* is allowed to vary from 0.1 to 0.4, with a step size of 0.1. This range for *sp* is selected to match to range of the proportion of DE features observed in real miRNA datasets [10-12].

To investigate the effect of left-censored measurements on the performance of the normalization methods, we create a simulation parameter *m*, which represents the proportion of measurements that are below detection limit. To generate datasets containing *m*% of missing values, we first obtain the *m*-*th* quantile expression level (*D*_*m*_) for each array, and then set measurements below *D*_*m*_ to missing for each array. *D*_*m*_’s are recorded individually for the downstream analysis. In this study, *m* is set to vary from 0 to 0.4, with a step size of 0.1, which is again chosen to reflect the range of missing values we encounter in real datasets. For each pair of sparsity parameter *sp* and missing parameter *m*, 20 replicate datasets are generated.

#### Normalization methods compared

In this simulation study, we compare the performance of the proposed Bayesian normalization method to the following widely used normalization methods: quantile normalization [18], variance stabilization normalization (VSN) [25], and no normalization.

#### Missing data imputation

For the Bayesian approach, imputation for the missing data below detection limit is automatically incorporated into the model framework, and no additional imputation work is necessary. For the other normalization methods investigated, the missing values were substituted with 0.5*DL, which is a common practice to handle data below DL [26,27].

#### Performance evaluation

The performance of different normalization methods is measured in terms of their capability of recovering truly differentially expressed features. For Bayesian normalization, features are ranked by the absolute value of the posterior mean of *δ*_*g*_, which is the winner's curse corrected effect size for each miRNA. For all the other normalization methods, data normalization is first performed, a moderated t-test [28] is then applied to the normalized data, and the features are subsequently ranked by the t-test p-values. The AUC (area under the receiver operating characteristic curve) is reported for each normalization method evaluated.

### Real data analysis

In addition to the simulation studies, we also compared the performance of various normalization methods on two sets of human tissue miRNA profiling data, which are microarray data and high resolution real-time RT-PCR (qRT-PCR) data.

#### Tissue microarray data

The human tissue microarray data were generated as part of an effort to compare between microarray and quantitative TaqMan qRT-PCR measurements [29]. The microarray dataset includes 43 samples hybridized on an Agilent Human miRNA Microarray 1.0 coming from nine different human tissues (brain, breast, heart, liver, placenta, testis, ovary, skeletal muscle, and thymus). There are four to five replicate arrays for each tissue type, and each array contains 534 miRNAs (excluding control probes). The microarray data are publicly available from GEO with series number GSE11879.

#### Tissue qRT-PCR data

**Figure 1:**
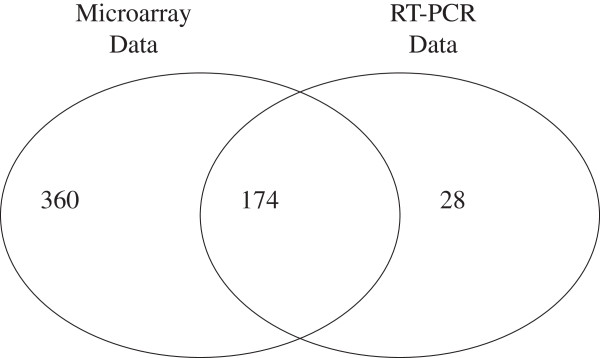
Venn diagram showing the relative distribution of miRNA probes between the human microarray miRNA profiling dataset (GSE11879) and the qRT-PCR dataset.

In a study on the processing patterns of miRNA, Lee et al. profiled the expression of 202 mature miRNAs using qRT-PCR from 22 different human tissues [30]. All the tissue types measured by Ach et al. are available in this data set except for breast tissue; and among the 534 miRNAs profiled in the microarray data, 174 miRNAs were also found in the qRT-PCR data (Figure 1). The availability of the high resolution qRT-PCR data provides us with high confidence the true fold change for a large number of miRNAs, which allows us to use fold changes for each miRNA (computed by the ratio of the expression between two tissues) from qRT-PCR data as an independent gold standard.

#### Normalization methods compared

As in the simulation study, we also compare the performance of quantile normalization, VSN, no normalization, and Bayesian normalization on the human tissue miRNA dataset. In addition, we also include the modified least-variant set (LVS) [15] in the set of methods being evaluated. LVS is a recently proposed normalization method that is specifically designed for miRNA data. Suo et al. have demonstrated that LVS outperforms no normalization, 75^th^ percentile-shift, quantile, global median, VSN, and LOWESS normalization methods in the same set of human tissue miRNA data as we use here [15].

#### Performance evaluation of normalization methods

We compare the performance of various normalization methods on the real data in terms of their ability to detect truly differentially expressed features, using the AUC metric.

In this human tissue dataset, different treatment conditions are essentially various human tissues, and truly differentially expressed features are those miRNAs that show distinct expression profiles between any pair of tissues. In order to compare the performance of various normalization methods in detecting truly differentially expressed hits, we adopted the same strategy as Suo et al. [15] in defining the set of true positive miRNAs. More specifically, differentially expressed miRNAs are selected as having qRT-PCR-based fold changes ≥ 3 (or ≤ 1/3) and P-values computed on the array data <0.01 (using the moderated t-test).

Since we have both qRT-PCR and microarray data available for eight tissues, AUCs for each normalization method on all 28 possible pairs of tissues will be computed.

## Results and discussions

### Performance evaluation of BHM normalization by simulation studies

**Figure 2:**
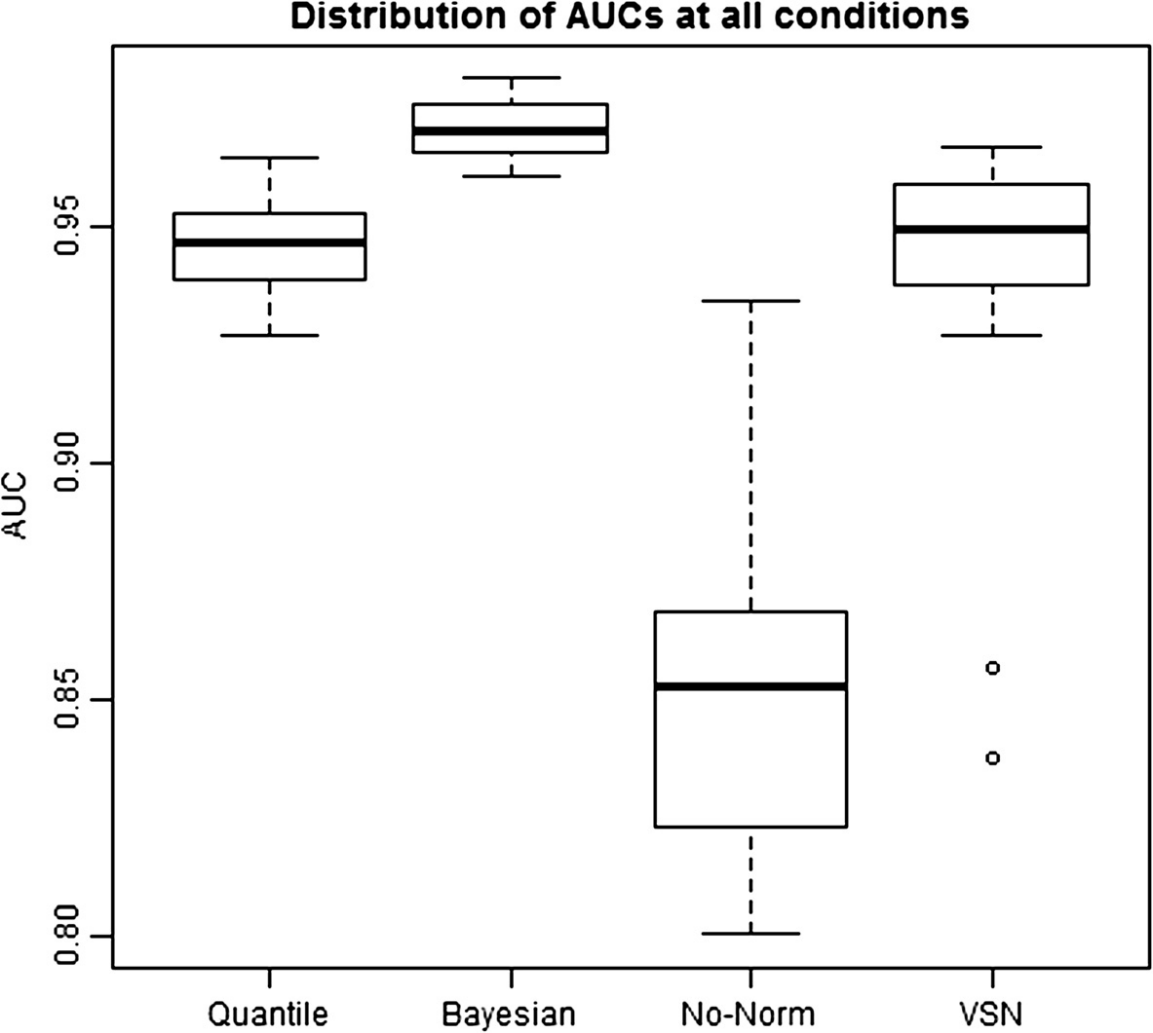
Boxplot showing the overall AUCs at all conditions for different normalization methods in simulation studies.

Our simulation studies are designed to closely examine the two unique features of miRNA datasets: (1) large proportion of differentially expressed features (i.e., parameter *sp*), and (2) large proportion of left-censored measurements due to detection limit (i.e., parameter *m*). For each unique pair of *sp* and *m*, the median AUC is obtained among the 20 replicates. The comparison of AUCs at all possible combinations of *sp* and *m* shows that the BHM method has the highest median value and the smallest variance among all of the normalization methods evaluated in this study (Figure 2). The high median AUC indicates that Bayesian normalization outperforms other normalization methods, and the small variance suggests that the superiority of the Bayesian approach to other methods is relatively consistent at a wide range of *sp* and *m*'s.

**Figure 3:**
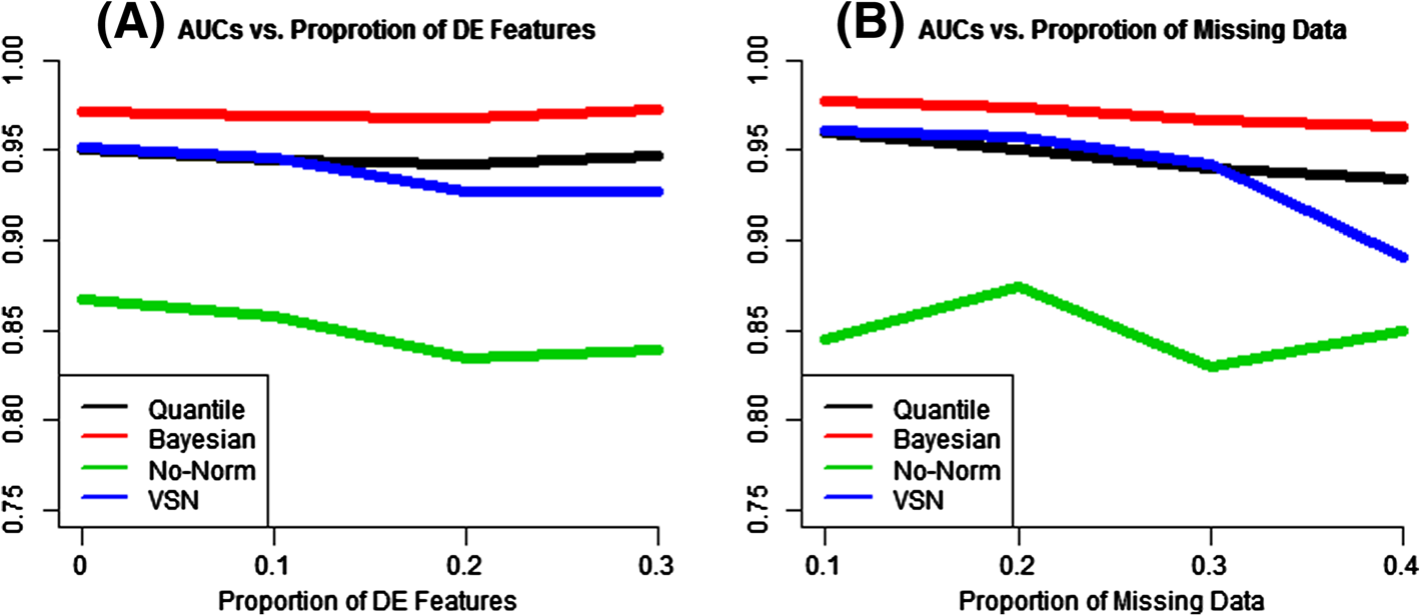
**AUC vs. Simulation Parameters of Interest. (A)** AUCs as functions of the proportion of differentially expressed (DE) features in simulation studies; **(B)**. AUCs as functions of the proportion of left-censored data in simulation studies.

We then summarize the relationship between AUC and the simulation parameters (*sp* and *m*) one at a time by plotting the median AUC (among replicates) versus the parameter of interest (Figure 3). We observe that the Bayesian approach yields a better AUC than all the other normalization methods all at ranges of *sp* (Figure 3a). Both VSN and quantile normalization outperform no normalization. Quantile normalization has a similar performance to that of VSN at small *sp*’s, but outperforms VSN when the proportion of differentially expressed features becomes larger than 10%. As *sp* increases, while there is a clear downward trend for the performance of VSN and no-normalization, the AUCs for the Bayesian approach and quantile normalization remain flat. These results suggest that the Bayesian framework and quantile normalization are robust against the variation of *sp*, while VSN and no-normalization are not.

When we examined the effect of the proportion of left-censored measurements on the performance of normalization methods, the Bayesian approach again consistently provides a better AUC than all the other normalization methods. When the data contain a small fraction of missing data, VSN appears to perform slightly better than quantile normalization; however, as the proportion of missing becomes larger, quantile normalization starts to outperform VSN. At all ranges of missing investigated in this simulation study, no-normalization is the worst performer among all the methods examined. In addition, we also observe that VSN is much more sensitive to the variation of *m* than the proposed Bayesian method (Figure 3b).

### Performance evaluation of BHM normalization by the analysis of tissue profiling data

The advantage of Bayesian normalization is further supported by the analysis of human tissue miRNA profiling data (GSE11879) using the qRT-PCR results [30] as the gold standard.

AUC is often used as a standard performance metric in method evaluation for many data mining applications; however, it practice, scientists may also be interested in the proportion of true positive features out of the declared positive features (i.e. precision). Therefore, in this part of the analysis, we use the average of AUC and AUPRC (area under the Precision-Recall curve) to evaluate the performance of different normalization methods.

**Figure 4:**
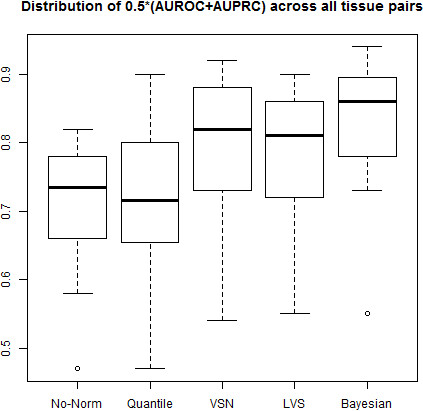
Distribution of (AUC+AUPRC)/2 for all 28 tissue pairs from the analysis of human miRNA profiling dataset (GSE11879) with different normalization methods using qRT-PCR results as the gold standard.

For all 28 pairs of human tissues, we plot the distributions of (AUC+AUPRC)/2 scores (Figure 4) and tabulate the number of times each normalization method yields the highest score among all the normalization methods (defined as Tmax), as well as the median score for each method (Table 1). Out of the 28 pairs of tissues analyzed, Bayesian normalization has the largest Tmax among all the methods compared. The median (AUC+AUPRC)/2 of LVS and VSN are similar, which are higher than quantile normalization and no normalization. We also noted that the variance of the (AUC+AUPRC)/2 distribution is the smallest for the Bayesian approach (Figure 4), which is in agreement with what we observed in the simulation studies, supporting the argument that the Bayesian approach is more robust than the other normalization methods examined.

**Table 1:** The number of times that each normalization method gives the highest (AUC+AUPRC)/2, and the median (AUC+AUPRC)/2 of each method for all 28 tissue pairs in the human miRNA profiling dataset (GSE11879) using qRT-PCR results as the gold standard

**Method**	**Bayesian**	**Quantile**	**No-Normalization**	**LVS**	**VSN**
T_max_	13	1	0	3	11
0.5*(AUC+AUPRC)_median_	0.86	0.72	0.74	0.81	0.82

An interesting finding from analyzing the human tissue miRNA data is that the performance of feature selection could actually be negatively impacted by inappropriately chosen normalization procedures. For instance, in the case of brain vs. liver comparison, we expect to find a large percentage of differentially expressed miRNAs. The (AUC+AUPRC)/2 scores are 0.79, 0.65, 0.82, 0.86, and 0.92 for no-normalization, quantile normalization, VSN, LVS, and Bayesian normalization respectively.

This scenario would violate the assumption that only a small proportion of features are expected to show differential expression in a microarray dataset. By comparing (AUC+AUPRC)/2 score of each method, we discover that normalization methods designed for mRNA data that tend to be overly aggressive (e.g., quantile normalization) provide virtually no additional benefits in recovering truly differentially expressed features. On the other hand, the performance of our BHM normalization remains robust.

## Conclusion

Profiling miRNA expression in cells with microarrays is becoming a widely used tool in elucidating miRNA functions. Normalization, often an overlooked aspect of data processing, is a critical step in the downstream detection of differentially expressed miRNAs. In the present study, we propose an integrative Bayesian approach to normalize miRNA data, and compare the performance of Bayesian method to other widely used miRNA normalization methods when the assumptions for mRNA normalization methods are violated.

Combining the findings from both simulation studies and the analysis of human tissue profiling data, it appears that normalization procedures can have a profound impact on the detection of truly differentially expressed miRNAs. Our Bayesian normalization framework appropriately addresses the left-censored nature of the miRNA microarray data (Figure 3b) and is robust against the variation of the proportion of differentially expressed features (Figure 3a). On the contrary, the other normalization methods evaluated do not seem to adequately handle these unique characteristics of miRNA data. In addition, we expect that the robust performance of our Bayesian approach can also benefit from the embedded winner's curse correction in our model and the reduction of the propagations of uncertainties (introduced from performing normalization, imputation, and feature extraction sequentially rather than in one integrative step).

Although our Bayesian method was formulated to handle normalization issues in miRNA data, we expect that biomarker discovery with other high-dimensional profiling techniques where there are a significant proportion of left-censored data points (e.g., proteomics) might also benefit from this approach.

## Abbreviations

miRNA: MicroRNA; DL: Detection limit; LVS: Least-variant set; BHM: Bayesian hierarchical modeling; DE: Differentially expressed; AUC: Area under the receiver operating characteristic curve; VSN: Variance stabilization normalization

## Competing interest

The authors declare that they have no competing interests.

## Authors’ contributions

JK conceived the study, implemented the Bayesian model, and drafted the manuscript. EX participated in the design of the study, and provided biological insights to the problems at hand. All authors have approved the final manuscript.

## Supplementary Material

Additional file 1 R package BMIRN that performs miRNA normalization described in this paper. Click here for file

Additional file 2 Instruction manual for the R package BMIRN. Click here for file
