# Supplementary material for: An integrated hierarchical Bayesian approach to normalizing left-censored microRNA microarray data
Source: BMC Genomics. 2013 Jul 26;14:507. doi: 10.1186/1471-2164-14-507 (PMC3734108; doi:10.1186/1471-2164-14-507)
Supplement: Additional file 2 — Instruction manual for the R package BMIRN. [file 1471-2164-14-507-S2.pdf]

Package 'BMIRN'

July 1, 2013

Type Package

Title An Integrated Hierarchical Bayesian Approach to Normalizing Left-Censored microRNA Microarray Data

Version 1.0

Date 2013-06-30

Author Jia Kang, Ethan Xu

Maintainer Jia Kang <jia.kang@merck.com>

Description This package uses a hierarchical Bayesian model to perform normalization on microarray miRNA data and calculates the posterior probability of a miRNA being differentially expressed.

License GPL-2

R topics documented:

BMIRN-package 1
alloc 2
datamat 3
det 4
DL 4
getDEprob 5

Index 7

`BMIRN-package` *BMIRN*

Description

This package uses a hierarchical Bayesian model to perform normalization on microarray miRNA data and calculates the posterior probability of a miRNA being differentially expressed.

Details

Package:	BMIRN
Type:	Package
Version:	1.0
Date:	2013-06-30
License:	GPL 2

Run function getDEprob to obtain the posterior probabilities of miRNAs being DE.

Author(s)

Jia Kang, Ethan Xu Maintainer: Jia Kang <jia.kang@merck.com>

References

An Integrated Hierarchical Bayesian Approach to Normalizing Left-Censored microRNA Microarray Data (Kang&Xu, 2013)

Examples

```
library(R2jags)
data(datamat)
data(DL)
data(alloc)
getDEprob(alloc=alloc,n.iter=2000,n.chains=1,n.thin=10,datamat=datamat,DL=DL)
```

`alloc` *Sample group allocation vector for datamat.*

Description

A sample allocation vector for the 10 arrays in the sample data file datamat. 0.5 and -0.5 denote case and control group (or vice versa), respectively.

Usage

```
data(alloc)
```

Format

The format is: num [1:10] -0.5 0.5 -0.5 -0.5 0.5 0.5 0.5 0.5 -0.5 -0.5

Details

Please use 0.5 and -0.5 to denote the two groups of samples.

Source

Simluation data.

References

NA

Examples

```
data(alloc)
```

---

datamat *Sample data file*

Description

Data file where rows are miRNAs, and columns are subjects. NA represents measurements below detection limit.

Usage

```
data(datamat)
```

Format

The format is: num [1:100, 1:10] 9.61 7.59 7.36 7.76 7.35 ... - attr(*, "dimnames")=List of 2 ..$: chr [1:100] "MIR_1" "MIR_2" "MIR_3" "MIR_4" ... ..$: NULL

Details

NA

Source

Simulation data.

References

NA

Examples

```
data(datamat)
```

`det` *Effect size of the miRNAs for the sample data datamat.*

Description

Effect size of the miRNAs for the sample data datamat. Negative/positive number represents down-regulated/upregulated miRNA respectively. 0 means the corresponding miRNA is not differentially expressed.

Usage

```
data(det)
```

Format

The format is: num [1:100] 0 0 0 0 0 0 0 0 0 0 ...

Details

NA

Source

simulation data

References

NA

Examples

```
data(det)
```

`DL` *Sample vector of array detection limits for the sample simulation data datamat.*

Description

Sample vector of array detection limits for the sample simulation data datamat.

Usage

```
data(DL)
```

Format

The format is: num [1:10] 6.39 6.62 6.46 5.6 7.08 ...

Details

NA

Source

Simulation data.

References

NA

Examples

```
data(DL)
```

---

getDEprob *Main function for data normalization and posterior probablity calculation*

Description

This function performs normalization on microarray miRNA data and calculates the posterior probability for miRNAs being differentially expressed.

Usage

```
getDEprob(alloc, n.iter, n.chains, n.thin, datamat, DL)
```

Arguments

`alloc`	Group allcation (e.g. case/control) vector for the arrays. use 0.5 for cases for -0.5 for controls. Please refer to data(alloc) for an example.
`n.iter`	Number of iterations used to update the mcmc chains. 1/2 of n.iter is used for burn-in.
`n.chains`	Number of mcmc chains.
`n.thin`	Number of thinning.
`datamat`	Data file where rows are miRNAs, and columns are subjects. Please use NA to represent measurements below detection limit.Please refer to data(datamat) for an example.
`DL`	A vector that contains the detection limit of the arrays. Please refer to data(DL) for an example.

Details

NA

Value

Returns the list of posterior probabilities that each miRNA is differentially expressed. miRNAs that have below-detection limit measurements across all subjects will be removed.

Note

NA

Author(s)

Jia Kang & Ethan Xu

References

An Integrated Hierarchical Bayesian Approach to Normalizing Left-Censored microRNA Microarray Data (Kang&Xu, 2013)

See Also

NA

Examples

```
library(R2jags)
data(datamat)
data(DL)
data(alloc)
getDEprob(alloc=alloc,n.iter=2000,n.chains=1,n.thin=10,datamat=datamat,DL=DL)
```

Index

∗Topic **datasets**
  alloc, 2
  datamat, 3
  det, 4
  DL, 4
∗Topic **optimize**
  getDEprob, 5
∗Topic **package**
  BMIRN-package, 1

alloc, 2

BMIRN *(*BMIRN-package*)*, 1
BMIRN-package, 1

datamat, 3
det, 4
DL, 4

getDEprob, 5
